# Αnti-prion effects of anthocyanins

**DOI:** 10.1016/j.redox.2024.103133

**Published:** 2024-03-28

**Authors:** Nikoletta Christoudia, Nikolaos Bekas, Eirini Kanata, Athanasia Chatziefsthathiou, Spyros Pettas, Korina Karagianni, Susana Margarida Da Silva Correia, Matthias Schmitz, Inga Zerr, Ioannis Tsamesidis, Konstantinos Xanthopoulos, Dimitra Dafou, Theodoros Sklaviadis

**Affiliations:** a Department of Genetics, Development and Molecular Biology, School of Biology, Aristotle University of Thessaloniki, 541 24, Thessaloniki, Greece; b Neurodegenerative Diseases Research Group, Department of Pharmacy, School of Health Sciences, Aristotle University of Thessaloniki, 541 24, Thessaloniki, Greece; c Department of Neurology, German Center for Neurodegenerative Diseases (DZNE), University Medicine Goettingen, 37075, Goettingen, Germany; d Department of Prosthodontics, School of Dentistry, Faculty of Health Sciences, Aristotle University of Thessaloniki, 541 24, Thessaloniki, Greece

**Keywords:** Prion, Anthocyanins, Anti-oxidant, Neuroprotection, Proteinopathies, PrP^Sc^

## Abstract

Prion diseases, also known as Transmissible Spongiform Encephalopathies (TSEs), are protein-based neurodegenerative disorders (NDs) affecting humans and animals. They are characterized by the conformational conversion of the normal cellular prion protein, PrP^C^, into the pathogenic isoform, PrP^Sc^. Prion diseases are invariably fatal and despite ongoing research, no effective prophylactic or therapeutic avenues are currently available. Anthocyanins (ACNs) are unique flavonoid compounds and interest in their use as potential neuroprotective and/or therapeutic agents against NDs, has increased significantly in recent years. Therefore, we investigated the potential anti-oxidant and anti-prion effects of Oenin and Myrtillin, two of the most common anthocyanins, using the most accepted in the field overexpressing PrP^Sc^*in vitro* model and a cell free protein aggregation model. Our results, indicate both anthocyanins as strong anti-oxidant compounds, upregulating the expression of genes involved in the anti-oxidant response, and reducing the levels of Reactive Oxygen Species (ROS), produced due to pathogenic prion infection, through the activation of the *Keap1-Nrf2* pathway. Importantly, they showcased remarkable anti-prion potential, as they not only caused the clearance of pathogenic PrP^Sc^ aggregates, but also completely inhibited the formation of PrP^Sc^ fibrils in the Cerebrospinal Fluid (CSF) of patients with Creutzfeldt–Jakob disease (CJD). Therefore, Oenin and Myrtillin possess pleiotropic effects, suggesting their potential use as promising preventive and/or therapeutic agents in prion diseases and possibly in the spectrum of neurodegenerative proteinopathies.

## Highlights

• Oenin and Myrtillin reduce ROS production in N2a22L cells.
• Oenin and Myrtillin activate the *Keap1-Nrf2* axis in N2a22L cells.
• Oenin and Myrtillin reduce PrP^Sc^ accumulation in N2a22L cells.
• Oenin and Myrtillin impede *de novo* PrP^Sc^ formation and aggregation.
• Oenin and Myrtillin are potent compounds for intervention against prion diseases.

## Graphical abstract

**Figure:**
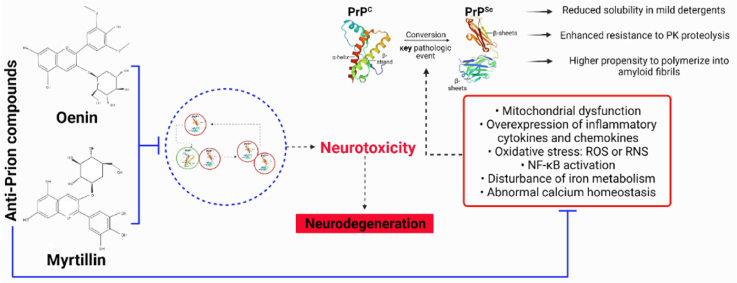

## Abbreviations

**NDs**: Neurodegenerative Disorders
**CJD**: Creutzfeldt-Jakob disease
**TSEs**: Transmissible Spongiform Encephalopathies
**PrP**^**C**^: Cellular prion protein
**PrP**^**Sc**^: Scrapie infectious prion protein
**ER**: Endoplasmic Reticulum
**ROS**: Reactive Oxygen Species
**OX0*B***: β-actin
**EDTA**: Ethylene Diamine Tetraacetic Acid
**PMSF**: Phenylmethylsulfonyl Fluoride
**PVDF**: Polyvinylidene difluoride
**RT-QuIC**: Real-time quaking-induced conversion
**Th-T**: Thioflavin – T
**AREs**: Antioxidant Response Elements
**AUC**: Area Under the Curve
**UPR**: Unfolded Protein Response

## 1 Introduction

Prion diseases are progressive and fatal Neurodegenerative Diseases (NDs), such as Creutzfeldt–Jakob disease (CJD), that affect humans and animals [[1], [2], [3]]. The fundamental event underlying scrapie infection seems to be a conformational change in the prion protein. Transmissible Spongiform Encephalopathies (TSEs) share a common pathogenic mechanism, which involves the autocatalytic conversion of the normal prion protein, PrP^C^, to its disease associated variant, PrP^Sc^. PrP^C^ molecules are repeatedly recruited and misfolded by PrP^Sc^, resulting in the formation of protease-resistant aggregates, known as amyloid fibrils [[1], [2], [3]]. Accumulation of PrP^Sc^ fibrils, results in Endoplasmic Reticulum (ER) stress, dysregulated calcium signaling, mitochondrial disfunction, and eventually neuronal cell death [[4], [5], [6]].

TSEs are strongly associated with oxidative stress [[7], [8], [9], [10]]. PrP^C^ has an important anti-oxidant function, binding bivalent metal ions [[11], [12], [13], [14]] and acting as a quencher of Reactive Oxygen Species (ROS) [[15], [16], [17]]. Loss of PrP^C^ activity leads to a marked increase in oxidation levels [8,[18], [19], [20], [21], [22], [23]]. Oxidative stress occurs early during the onset and the progression of prion diseases [20,24], and models with compromised anti-oxidation response showcase accelerated disease progression [25,26]. Conversely, reduced ROS levels are associated with increased survival and improved phenotype [22]. Furthermore, PrP^Sc^ aggregation causes an uptake in mitochondrial ROS production and decreased levels of oxidative phosphorylation (OXPHOS) [[27], [28], [29]], which further aggravates ROS formation and oxidative stress observed in prion-affected cells [23,30].

Enhancement of anti-oxidant responses emerges as a promising therapeutic approach against neurodegeneration [31,32]. Anthocyanins (ACNs) are polyphenolic derivatives of the anthocyanidin flavonoid group, and act as water soluble vacuolar pigments in various flowers, fruits and vegetables (Supplementary Fig. 1) [33]. Due to their potent anti-oxidant action, ACNs have been extensively tested as potential remedies against oxidative stress associated conditions, such as cancer [[34], [35], [36], [37]], cardiovascular diseases [[38], [39], [40], [41]] and neurodegeneration [[42], [43], [44], [45]]. Importantly, their ability to penetrate the Blood-Brain Barrier (BBB) renders them exceptional neuroprotective compounds [46].

Oenin (Malvidin-3-glucoside) and Myrtillin (Delphinidin 3-glucoside) are two of the most prevalent ACNs present in grapes and red wine [47]. While, they have previously showcased anti-oxidant and anti-inflammatory activity [[48], [49], [50], [51], [52], [53]], their potential effect in prion diseases has not yet been investigated. In this study, the anti-prion potential of Oenin and Myrtillin (Supplementary Fig. 2) is described for the first time. In scrapie-infected murine neuroblastoma N2a (ScN2a) cells, we addressed the effect of ACNs for the reduction of ROS levels through the activation of the *Keap1-Nrf2* pathway, and the reduction of PrP^Sc^ aggregates in ScN2a22L cells, and also the inhibition of the formation of PrP^Sc^ fibrils in the Cerebrospinal fluid (CSF) of CJD patients. Therefore, our results highlight the strong potential of Oenin and Myrtillin against prion diseases and possibly other neurodegenerative proteinopathies.

## 2 Materials and methods

### 2.1 Cell culture and LD_50_ Estimation

The murine neuroblastoma cell line N2a22L has been utilized, in which the murine scrapie prion 22L strain is expressed, leading to the sustained production of Proteinase K (PK) resistance protein, PrP^Sc^ [54]. The N2a22L cell line has been widely used over the years as the most reliable model for the study of PrP^Sc^ aggregation [18,[55], [56], [57], [58], [59], [60], [61], [62], [63], [64]]. Cells were cultured in Opti-MEM (51985042, Invitrogen Waltham, MA, USA) supplemented with 10% Fetal Bovine Serum (FBS) under a 5% CO_2_ at 37 °C. Oenin (0911S, Extrasynthese Genay, France) and Myrtillin (0938S, Extrasynthese Genay, France) were dissolved in dimethyl sulfoxide (DMSO). Lethal Dose 50% (LD_50_) values were estimated using the 3-(4,5-dimethylthiazol-2-yl)-2,5-diphenyl-2H-tetrazolium bromide (MTT) Assay (M6494, Invitrogen, Waltham, MA, USA), following 48-h incubation [65,66] with gradually increased concentrations of Oenin and Myrtillin. Control cells were treated with DMSO in concentrations matching those delivered with the compounds. All experiments were performed in triplicates.

### 2.2 *In vitro* estimation of ROS amounts

N2a22L cells were incubated with Oenin or Myrtillin (250 μΜ) for 48 h. Cells were incubated for 30 min at room temperature with H_2_DCFDA (2′,7′-Dichloro-dihydro-fluorescein, D399, Invitrogen, Waltham, MA, USA) dissolved in DMSO, and fluorescence was measured using a Tecan fluorometer. Controls received DMSO at concentrations matching those delivered with the compounds. The same set of experiments were performed after pre-treatment with two different concentrations of H_2_O_2_, 6.25 μM and 200 μM for 30 min before addition of Oenin and Myrtillin. Analysis was done in triplicates and relative fluorescence was expressed as “% of maximum emission”, determined with Tecan Magellan software (https://lifesciences.tecan.com/software-magellan, accessed December 19, 2023).

### 2.3 RNA Isolation and qPCR

Total RNA was extracted using spin columns (740955.250, Macherey Nagel, Dueren, Germany). For cDNA synthesis, 500 ng total RNA and the TaKaRa PrimeScript RT Reagent Kit (RR037A, TAKARA, Shiga, Japan) were used. Relative expression of Heme Oxygenase 1 (*HMOX1*), Glutamate Cysteine Ligase Regulatory Subunit (*GCLM*) and Nuclear factor (erythroid – derived 2) - like 2 (*NFE2L2*) was estimated by qPCR, using β-actin (*ACTB*) for normalization. The KAPA SYBR fast qPCR kit, 20 ng cDNA and 0.1 μΜ each primer was used (Supplementary Table 1). Reactions were performed in a 7500 Fast Real-time PCR System (Applied Biosystems), in triplicates.

### 2.4 *In vitro* assessment of PrP^Sc^ aggregation

Oenin-, Myrtillin-treated and control N2a22L cells were lysed in ice-cold lysis buffer (10 mM Tris pH 7.5, 100 mM NaCl, 10 mM EDTA, 0.5% v/v Triton-X-100, 0.5% w/v sodium deoxycholate) and centrifuged (1 min, 14,000×*g*). Total protein in the supernatant was estimated with Bradford reagent (A6932, 0250, AppliChem, Darmstadt, Germany). One fraction of each lysate was digested with PK (1.24569.0100, Merck, Darmstadt, Germany) in 1% w/v N lauryl-sarcosine. Phenylmethylsulfonyl Fluoride (PMSF) (5 mM final concentration) was used to stop the reaction. PK treated samples (PK+) and non-PK treated samples (PK-) were resolved on 12% w/v poly-acrylamide gels, electro-transferred onto Polyvinylidene Fluoride (PVDF) membranes and subjected to Western Blot analysis using the monoclonal antibody 6H4. Chemiluminescence was used for development on X-ray films. Films were digitized and relative protein levels were estimated with ImageJ (available at https://imagej.net/ij/index.html, accessed on May 08, 2023), utilizing exposures within the linear dynamic range of the film.

For each sample, the ratio of the intensity of PrP-immunopositive bands in the PK-resistant fraction (PK+) to the intensity of total PrP in the non-PK (PK-) treated fraction was estimated and expressed relative to controls using the formula:

[PrP(_RES_)ACN/ PrP(_TOT_)_ACN_]*100/[PrP(_RES_)Cntr/PrP(_TOT_)Cntr]

Where PrP(_RES_)ACN and PrP(_TOT_)ACN are the intensity of PrP bands in the PK-treated and non-PK-treated fractions respectively in Oenin or Myrtillin treated samples. PrP(_RES_)Cntr and PrP(_TOT_)Cntr show the intensity of PrP bands in the PK-treated and non-PK treated controls, respectively. In order to verify that PK treatment conditions resulted in complete digestion of PrP^C^, cell lysates from N2a58 cells that were not prion-infected underwent a similar PK treatment and were subsequently immunolabelled for PrP. PK treatment completely digested PrP^C^, because no PrP immunoreactivity was found in these experiments (Supplementary Fig. 4). The immunoreactive bands detected in N2a22L cells that were treated with PK do not correspond to partially digested PrP^C^, but rather to PrP^Sc^.

### 2.5 Cell free detection of *de novo* PrP^Sc^ fibrillation through RT-QuIC

Real-time quaking-induced conversion reactions, RT-QuIC [67] were performed using CSF containing PrP^Sc^ seed material from patients with confirmed sCJD diagnosis, originating from the National Reference Center for TSEs, Göttingen, Germany. 15 μL CSF (diluted 1000 times) was mixed with 85 μL reaction buffer (5 × PBS pH 6.9, 170 mM NaCl, 1 mM EDTA, 10 μM Thioflavin-T and 0.1 mg/mL recPrP^C^). Oenin or Myrtillin were added to a final concentration of 2.5 μM. Reactions were set in 96-well black bottom optical plates and carried out in a BMG Labtech FluoO Star OPTIMA plate reader at 42 °C for 80 h with intermittent rest and shaking cycles. Thioflavin-T (Th-T) fluorescence was measured every 30 min. Analysis was performed in triplicates.

### 2.6 Statistical analysis

GraphPad Prism version 8.0.2 for Windows (GraphPad Software, San Diego, CA, USA, www.graphpad.com, Accessed on May 08, 2023) was used. Non-linear regression analysis was applied to the dose-response equations for LD_50_ determination. Differences in gene expression and PrP^Sc^ accumulation between untreated and treated cells were estimated with unpaired, one-tailed T-tests. Data represent Standard Error of Mean (SEM) of three independent experiments and P-values of 0.05 or lower were considered statistically significant.

## 3 Results

### 3.1 Assessment of LD_50_ of Oenin and Myrtillin in N2a22L cell line

The viability assay showed Oenin as less toxic. Oenin LD_50_ values estimated at 506.8 μM as opposed to Myrtillin LD_50_ estimated at 293.1 μM (Supplementary Fig. 3). A concentration of 250 μM for each ACN was used for the rest of the study, in which both compounds presented no cytotoxicity to N2a22L cells, and cell treatment entailed 48-h incubation.

### 3.2 Oenin and Myrtillin reduced ROS levels in ScN2a22L cells

**Fig. 1:**
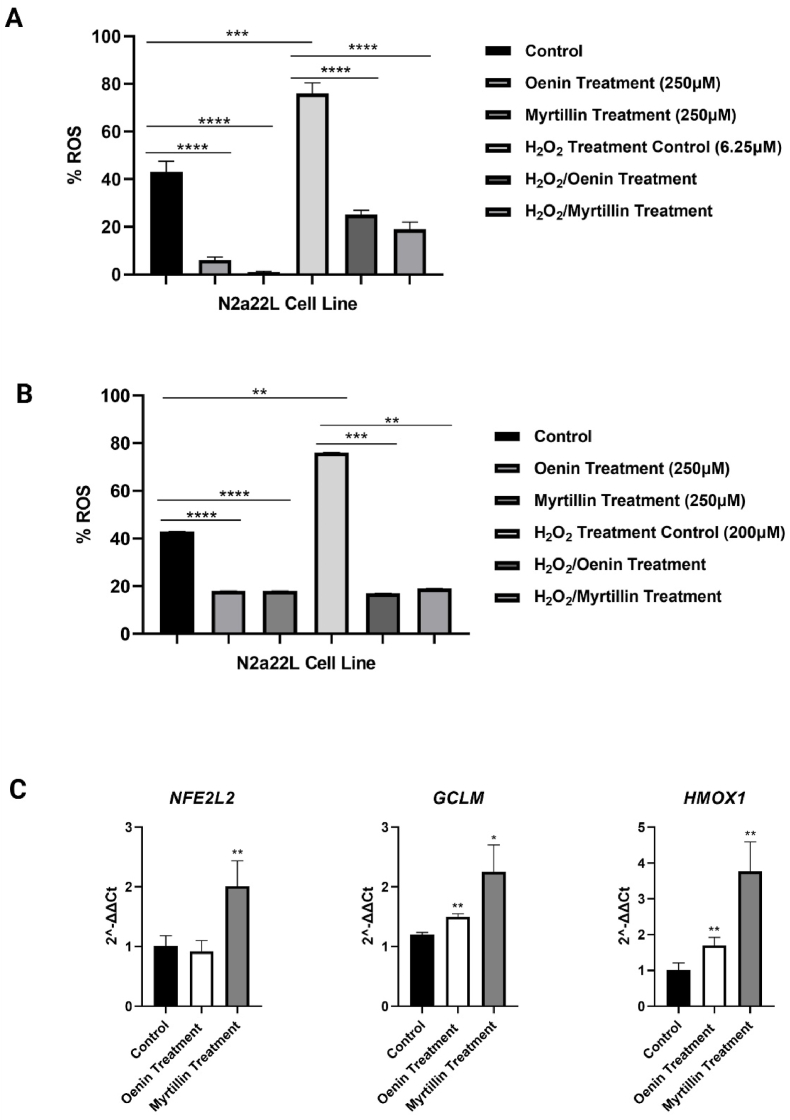
Anti-oxidant effects of Oenin and Myrtillin in N2a22L cells. ROS levels were measured in N2a22L cells after a 48-h treatment with Oenin or Myrtillin (250 μΜ each), without or following pre-treatment with **(A)** 6.25 μM H_2_O_2_ and **(B)** 200 μM H_2_O_2_, to induce oxidative stress. Controls received DMSO at concentrations matching those delivered with the compounds. For ROS measurement, H_2_DCFDA, dissolved in DMSO, was added in the cell medium at a final concentration of 20 μΜ and cells were further incubated for 30 min at room temperature. Then, fluorescence was measured using a Tecan fluorometer. The % ROS was calculated based on the maximum ROS production value. **(C)** Oenin and Myrtillin induce the expression of *Keap1-Nrf2* pathway gene targets in N2a22L cells. The *Keap1-Nrf2* pathway is a key cellular defense mechanism against oxidative stress, that protects cells by reducing the risk of ROS-mediated damage through the activation of cytoprotective enzymes. More specifically, Nrf2 binds to Antioxidant Response Elements (AREs) in the promoters of anti-oxidant genes, aiming to restore redox homeostasis [68]. The expression of *NFE2L2* (encoding Nrf2), *GCLM* and *HMOX1* in Oenin and Myrtillin treated cells (250 μΜ, 48 h) is assessed in N2a22L cells, relative to controls (administered DMSO at the same concentration as those delivered with the compounds). Data represent Standard Error of Mean (SEM) of three independent experiments. Stars denote statistical significance (unpaired, one-tailed, T-test); *: p-value <0.05, **: p-value <0.01.

Prion diseases are associated with elevated oxidation and ROS production [[8], [9], [10],^22^]. Owing to their known anti-oxidant activity [41,48], it was tested whether Oenin and Myrtillin could affect ROS levels in ScN2a22L cells. Both compounds significantly reduced the endogenous ROS levels and the ROS produced after H_2_O_2_ administration (Fig. 1). It is worth noting that, Myrtillin in most cases (without H_2_O_2_ administration and with 6.25 μM) neutralized better the amount of generated ROS when compared with Oenin. These results showcased that Oenin and especially Myrtillin have strong anti-oxidant action in prion affected cells.

### 3.3 Oenin and Myrtillin activate the *Keap1-Nrf2* pathway

Many compounds exert their anti-oxidant neuroprotective effects through *Keap1-Nrf2* activation [[69], [70], [71], [72], [73], [74], [75], [76]]. Consequently, it was tested whether the observed anti-oxidant effects of Oenin and Myrtillin in N2a22L cells also depended on *Keap1-Nrf2* induction. Indeed, both ACN compounds managed to trigger the expression of key Nrf2 antioxidant target genes, *GCLM* and *HMOX1* [77]. Interestingly, Myrtillin not only presented a more profound effect in *GCLM* and *HMOX1* levels, but also upregulated the expression of Nrf2 (*NFE2L2*) (Fig. 1).

### 3.4 Oenin and Myrtillin decrease the levels of PrP^Sc^ aggregates

**Fig. 2:**
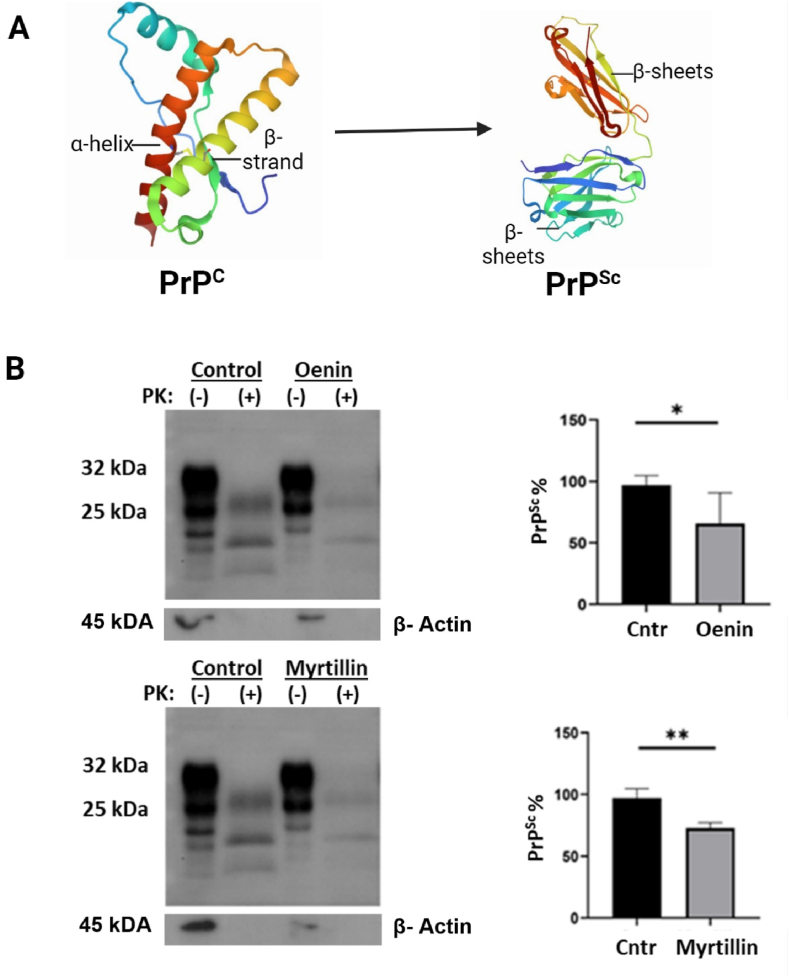
Oenin and Myrtillin reduce PrP^Sc^ aggregation in N2a22L cells. **(A)** Representation of the structural rearrangement taking place during the PrP^C^–PrP^Sc^ conversion. During this process, the α-helix rich PrP^C^ (in which C stands for the cellular form of the normal prion protein and is expressed in neurons and the spinal cord) is transformed into the β-sheet enriched PrP^Sc^ (Sc stands for scrapie, the prion disease of sheep and goats). This results in physio- and bio-chemical properties distinct from PrP^C^, including reduced solubility in mild detergents, enhanced resistance to partial proteolysis by PK. **(B)** Representative Western blot results for each compound, along with the densitometric analysis from three independent experiments are depicted. Cell lysates from Oenin and Myrtillin treated N2a22L cells as well as controls (administered DMSO at the same concentrations as those delivered to the ACN treated cells) were processed for PrP immunodetection. A fraction of each lysate (150 μg total protein) was treated with proteinase K (PK+, 1.25 μg PK/mg total protein) for 1 h at 37 °C, to allow the identification of the partially resistant to PK, PrP^Sc^. Due to its conformation, PrP^Sc^ is not accessible for enzymatic treatment, except a segment at its amino-terminal site which is digested resulting in the characteristic band shift of PrP immunopositive bands towards lower molecular weights. Analysis of non-PK treated (PK-) material (50 μg) from the same sample allowed total PrP detection (PrP^C^ and PrP^Sc^). For PrP immunodetection the monoclonal 6H4 antibody (7500997, Invitrogen, Waltham, MA, USA) was used (0.2 μg/mL). PK activity degrades β-Actin, thus it is not visible in PK(+) samples. Blots were developed on autoradiography films using chemiluminesence. Densitometric analysis was performed with ImageJ. Bar graphs show the conversion rate of each ACN treated sample (PrP^Sc^/Total PrP) relative to the control conversion rate (PrP^Sc^ %). Data represent Standard Error of Mean (SEM). Stars denote statistical significance (unpaired, one-tailed, T-test); *: p value < 0.05, **: p value < 0.01.

The PrP^Sc^ leads to enhanced resistance against PK and higher propensity to polymerize into amyloid fibrils, the primary cause of prion diseases [[78], [79], [80], [81]]. Consequently, reducing the amount of PrP^Sc^ is of paramount importance for any potential anti-prion and neuroprotective compound. For that reason, the ability of Oenin and Myrtillin to increase the PK sensitivity of PrP^Sc^ aggregates, was tested. Both compounds significantly reduced the amount of PrP^Sc^ aggregation in N2a22L cells, providing further support for their potential anti-prion action (Fig. 2).

### 3.5 Oenin and Myrtillin inhibit the *de novo* PrP^Sc^ aggregation

**Fig. 3:**
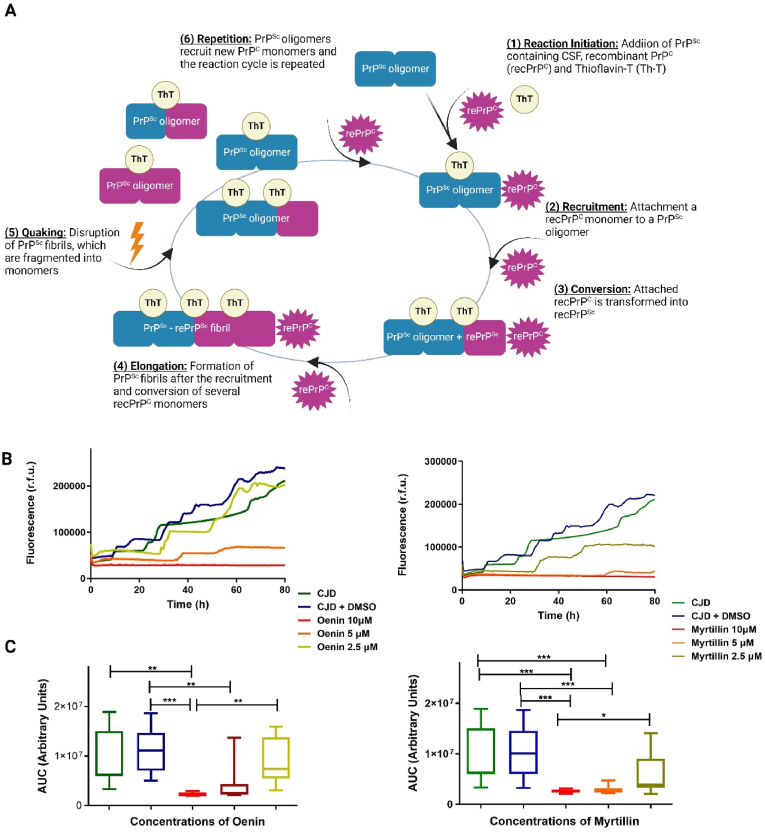
Oenin and Myrtillin inhibit recPrP^C^ fibrillation in RT-QuIC assays seeded with human PrP^Sc^. **(A)** Summary of RT-QuIC steps: **(1)** A sample containing PrP^Sc^ (such as CSF from CJD patients) is mixed with a recombinant PrP^C^ (recPrP^C^) monomers and Th-T, which specifically binds to β-sheets, leading to fluorescence. **(2)** The recPrP^C^ monomers are recruited by the PrP^Sc^ oligomers. **(3)** Recruited recPrP^C^ monomers are transformed into recPrP^Sc^ and the PrP^Sc^ oligomers are elongated. **(4)** Creation of PrP^Sc^ -recPrP^Sc^ fibrils. **(5)** Quaking induces fragmentation of the PrP^Sc^ fibrils. **(6)** The process is repeated [83,91,92]. **(B)** Aggregation of recPrP^C^ in RT-QuIC was assessed in the CSF from twelve different CJD patients. Oenin and Myrtillin were added in the reaction mixture of RT-QuIC in three different concentrations (2.5, 5 and 10 μM) and the results were compared with that from CSF only and CSF with DMSO. Reactions were set using 15 μL of diluted seed material and performed at 42 °C for 80 h with intermittent rest and shaking cycles. Th-T fluorescence, as a measure of protein aggregation, was recorded every 30 min. The graph depicts combined (mean) data from the results acquired from the twelve independent patients CSFs used as seed. sCJD: positive control; RT-QuIC assays performed with no anthocyanin supplementation. Oenin, Myrtillin: RT-QuIC assays performed in the presence of Oenin or Myrtillin. Both compounds block PrP aggregates formation. **(C)** Quantification of Oenin and Myrtillin effects on PrP conversion and aggregation inhibition. Box plots represent the Standard Error of Mean (SEM) of the Area Under Curve (AUC) calculated for the individual fluorescence curves of each replicate reaction. AUC values were used as a measure of protein conversion and aggregation. Stars indicate statistical significance (unpaired, one-tailed, T-test). **: p value < 0.01, ***: p value < 0.001.

Oenin and Myrtillin promoted the clearance of PrP^Sc^ aggregates. As a result, it was tested whether they could also block the *de novo* PrP^Sc^ fibrillation. For that purpose, RT-QuIC [82], a highly sensitive technique that is routinely used for the diagnosis of prion diseases and similar neurodegenerative disorders, that it is able to detect the presence of misfolded proteins with almost 100% accuracy, was utilized [[83], [84], [85]]. Consequently, it has also been deployed for the screening of anti-prion compounds [67,[86], [87], [88], [89], [90]]. Both compounds showcased remarkable anti-aggregation capacity, as they completely inhibited the formation of PrP^Sc^ fibrils at concentrations of 5 and 10 μM for both compounds, and Myrtillin maintained moderate anti-aggregation action even at 2.5μΜ (Fig. 3).

## 4 Discussion

Prion diseases belong to a group of NDs known as proteinopathies, or prion-like diseases, in which pathologic protein misfolding and accumulation, plays a crucial role in disease development and progression [[93], [94], [95], [96]]. In the case of prion diseases, this is due to the transformation of normal prion protein, PrP^C^, into the pathologic PrP^Sc^ [78,79]. In this study, the strong anti-prion ability of Oenin and Myrtillin are described for the first time. Treatment with Oenin and Myrtillin for just 48 h was able to significantly decrease the number of PrP^Sc^ aggregates in N2a22L cells. Additionally, both compounds completely inhibited the *de novo* formation of PrP^Sc^ fibrils in the CSF of CJD patients, at a concentration of 10 μM and 5 μM, in the case of Myrtillin, maintained robust anti-prion action even at 2.5 μM.

While the mechanism of their anti-aggregation action is yet to be elucidated, the *Keap1-Nrf2* pathway is highly likely to be responsible. Indeed, previous studies in proteinopathies showed that Nrf2 activation inhibited the formation and/or reduced the number of existing aggregates of α-synuclein [[97], [98], [99], [100]], amyloid beta [[101], [102], [103], [104]] and tau [[105], [106], [107]], whereas Nrf2 deficiency promotes protein aggregation [[108], [109], [110]]. The Nrf2 transcription factor has long been identified as a modulator of autophagy [[111], [112], [113], [114]], and is also associated with the Unfolded Protein Response (UPR) [100,[115], [116], [117], [118], [119], [120]], which might explain its ability to reduce pathologic protein aggregation in several proteinopathies, including prion diseases [121,122]. Interestingly, activation of the UPR has been utilized as a potential treatment against CJD [123], whereas p62 mediated Nrf2 activation and subsequent upregulation of autophagy levels has been proposed as a therapeutic strategy for prion diseases [6].

Additionally, Oenin and Myrtillin could be directly interacting with the prion protein. Indeed, other flavonoids are capable of directly binding to PrP^C^. For example, Quercetin, interaction with PrP^Sc^ fibrils renders them vulnerable to protein degradation, leading to de-aggregation [123,124]. Moreover, Apigenin and Nepetin managed to inhibit the fibrillation of the PrP_106-126_ peptide and also depolymerize the already formed fibrils [125]. It is also worth noting that, Oenin and Myrtillin might exert their anti-prion action with a combination of different mechanisms. Quercetin can simultaneously bind to the C-terminal region of murine prion protein and also act as an anti-oxidant [124,126]. A similar observation was made with Curcumin, which can bind to PrP^Sc^ fibrils, as well as intermediate aggregates of the PrP^C^–PrP^Sc^ conversion, while also exerting anti-oxidant action [127,128].

Oxidative stress has been identified as a hallmark of neurodegeneration [32,[129], [130], [131]]. In accordance with other anti-oxidant compounds exhibiting anti-prion activity [72,124,126,128,132,133], Oenin and Myrtillin successfully decreased the levels of ROS in prion-infected cells. While Oenin didn't affect Nrf2 expression levels, it managed to activate the *Keap1-Nrf2* pathway (albeit less effectively compared to Myrtillin). Indeed, the important step for the activation of *Keap1-Nrf2* target genes is nuclear translocation of Nrf2 [70,73,75,114,134,135]. As a result, one potential explanation for the activity of Oenin is that it successfully triggered the nuclear translocation of Nrf2, but due to the fact that it didn't lead to upregulation of Nrf2 itself, the activation of the *Keap1-Nrf2* pathway was less potent.

## 5 Conclusions

**Fig. 4:**
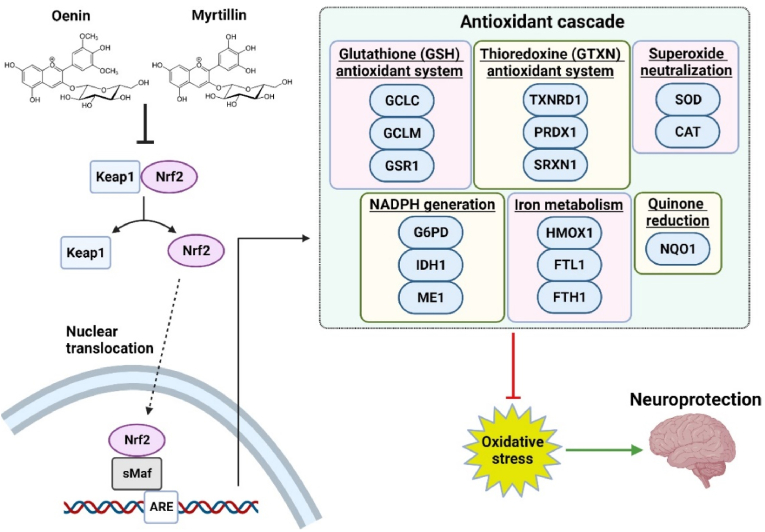
Oenin and Myrtillin protect cells from PrP^Sc^ mediated oxidative stress through *Keap1-Nrf2* activation. Treatment with either, Oenin or Myrtillin, disrupts the *Keap1/Nrf2* dimer, resulting in Nrf2 nuclear translocation. This leads to the activation of a series of anti-oxidant genes, that are related to the glutathione and thioredoxin anti-oxidant systems, NADPH regeneration, iron metabolism, quinone reduction and superoxide neutralization [75,[135], [136], [137]]. Consequently, excessive ROS production is inhibited, and cellular homeostasis is restored, inducing neuroprotection.

To summarize, our results of the current study provide promising evidence regarding the anti-prion neuroprotective potential of Oenin and Myrtillin. Both compounds are able to de-aggregate pre-existing PrP^Sc^ fibrils, and also severely inhibit the process of *de novo* PrP^Sc^ fibrillation. Moreover, they acted as potent anti-oxidants, decreasing ROS levels through the activation of the *Keap1-Nrf2* pathway, leading to neuroprotection (Fig. 4).

## Funding

This work was supported by the European Regional Development Fund, 2021–2023, Investment Research Plans for Business Research and Development of Central Macedonia (Grant number: KMP6-0079465).

## Financial interests

The authors declare they have no financial interest.

## Non-financial interests

None.

## CRediT authorship contribution statement

**Nikoletta Christoudia:** Writing – original draft, Visualization, Validation, Software, Methodology, Investigation, Formal analysis, Data curation, Conceptualization. **Nikolaos Bekas:** Writing – original draft, Visualization. **Eirini Kanata:** Writing – review & editing, Writing – original draft, Methodology, Investigation, Data curation. **Athanasia Chatziefsthathiou:** Visualization, Methodology, Data curation. **Spyros Pettas:** Visualization, Methodology, Data curation. **Korina Karagianni:** Visualization, Methodology, Data curation. **Susana Margarida Da Silva Correia:** Visualization, Software, Methodology, Data curation, Conceptualization. **Matthias Schmitz:** Writing – review & editing, Resources. **Inga Zerr:** Writing – review & editing, Resources, Conceptualization. **Ioannis Tsamesidis:** Software, Methodology, Data curation, Conceptualization. **Konstantinos Xanthopoulos:** Writing – review & editing, Supervision, Resources, Funding acquisition. **Dimitra Dafou:** Writing – review & editing, Supervision, Resources, Funding acquisition, Conceptualization. **Theodoros Sklaviadis:** Writing – review & editing, Supervision, Resources, Funding acquisition.

## Declaration of competing interest

The authors declare that they have no known competing financial interests or personal relationships that could have appeared to influence the work reported in this paper.

## Data Availability

No data was used for the research described in the article.
