# Supplementary material for: Αnti-prion effects of anthocyanins
Source: Redox Biol. 2024 Mar 28;72:103133. doi: 10.1016/j.redox.2024.103133 (PMC10990977; doi:10.1016/j.redox.2024.103133)
Supplement: Multimedia component 1 [file mmc1.docx]

**Supplementary Data**


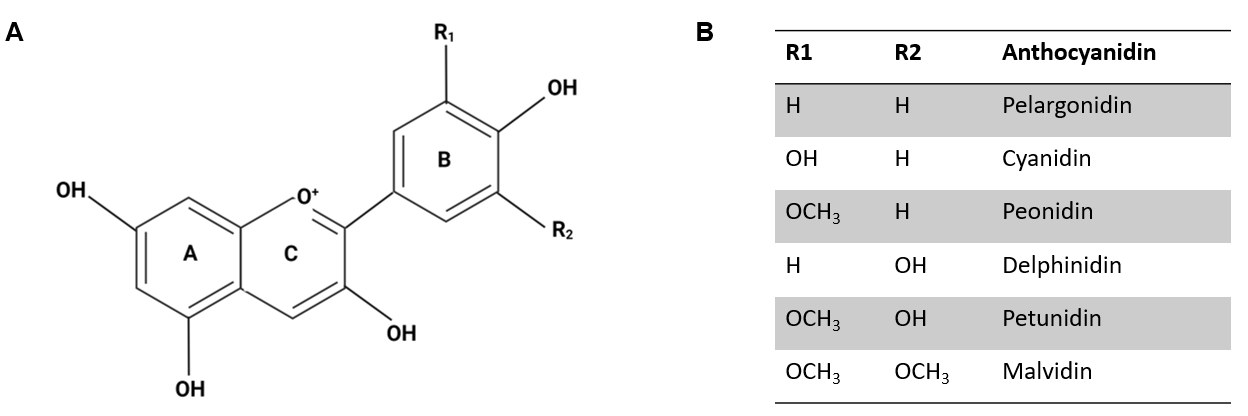


**Supplementary Figure 1:** (**A**) General flavonoid structure. Flavonoids possess a characteristic three-ring structure, which is conserved across all family members. Several classes of flavonoids exist, including Anthocyanins (ACNs), which differ depending on substitutions of the A-, B-, and C- rings. (**B**) Structure of the six most common anthocyanidins in nature with the differentiations on R1 and R2.

**
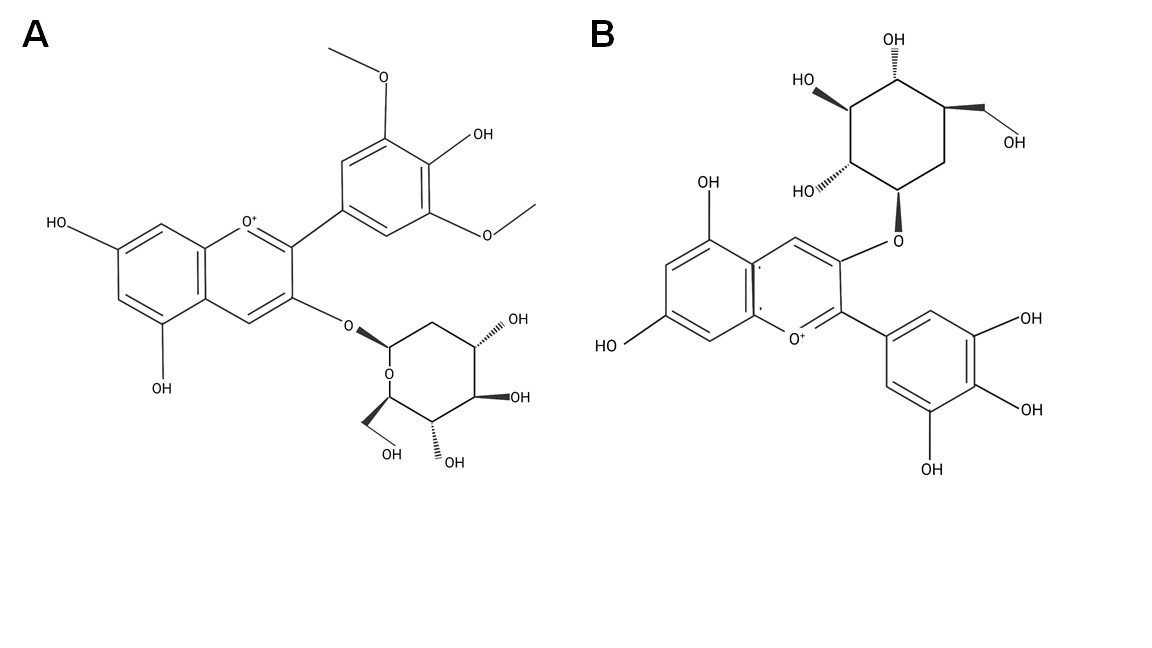
**

**Supplementary Figure 2:** Chemical structures of (**A**) Oenin (MW: 528.89 g/mol) and (**B**) Myrtillin (MW: 500.84 g/mol).

**Supplementary Table 1:** Primer sequences.

| **Primer Name** | **Sequence (5’- 3’)** |
| --- | --- |
| *HMOX1-F*orward | CAG TCG CCT CCA GAG TTT CC |
| *HMOX1-Reverse* | TAC AAG GAA GCC ATC ACC AGC |
| *GCLM-F*orward | CTG CAA AAC TGT TCA TTG TAG G |
| *GCLM-Reverse* | CTA TTG GGT TTT ACC TGT G |
| *NFE2L2-F*orward | GCA ACT CCA GAA GGA ACA GG |
| *NFE2L2-Reverse* | GTG GGC AAC CTG GGA GTA G |
| *ACTB-F*orward | CAG CTT CTT TGC AGC TCC TT |
| *ACTB- Reverse* | CAC GAT GGA GGG GAA TAC AG |


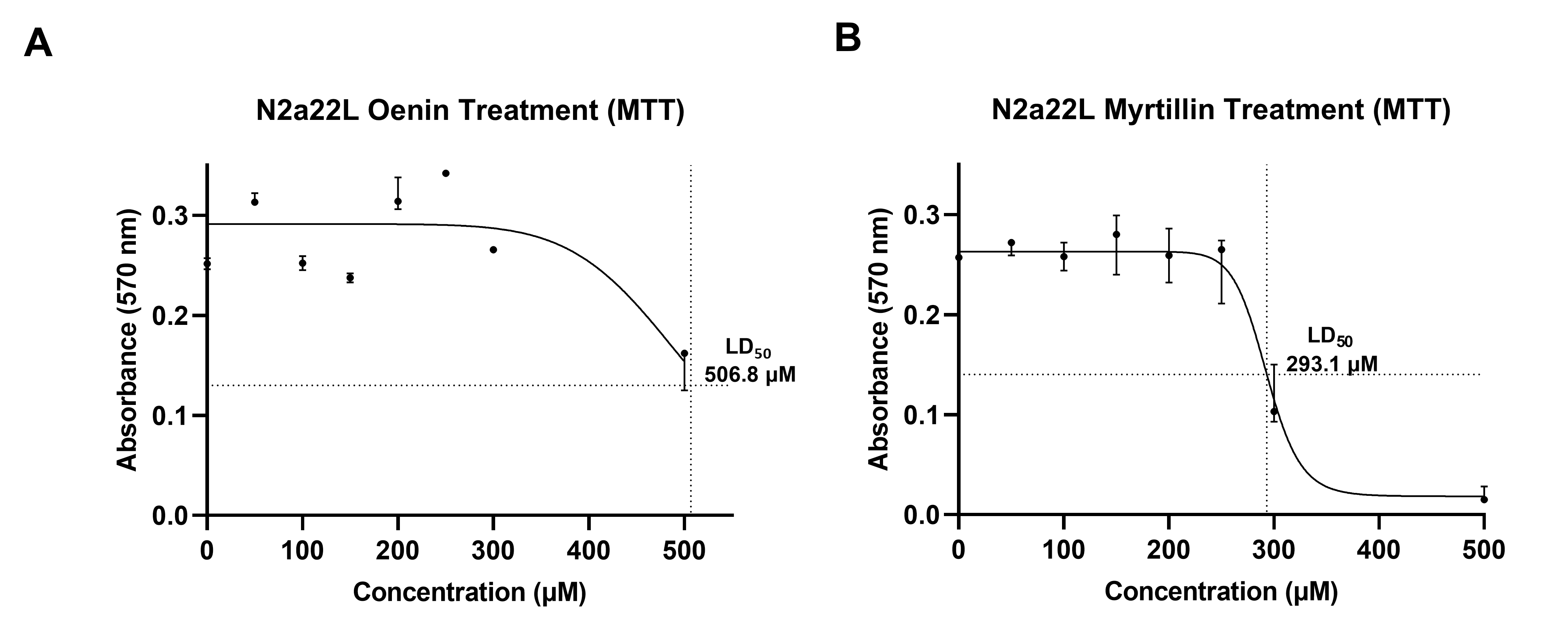


**Supplementary Figure 3:** Cell Viability Assessment by means of the 3-(4,5-dimethylthiazol-2- yl)-2,5-diphenyl-2H-tetrazolium bromide (MTT) Assay for **(A)** Oenin and **(B)** Myrtillin following 48 h incubation in N2a22L cell line. Control cells were administered DMSO in concentrations matching those delivered with the compounds. Graphs depict the absorbance at 570 nm determined for N2a22L cell line following treatment with the indicated compounds at gradually increasing concentrations. The background absorbance of the plates at 630 nm was also measured and subtracted from 570 nm measurement. LD_50_ values were estimated for each compound based on non-linear regression analysis for curve fitting using the GraphPad software (v 8.0.2). The black dots in graphs for **(A)** Oenin and **(B)** Myrtillin represent the fitted curve in each case; determined LD_50_ values are depicted in each graph.


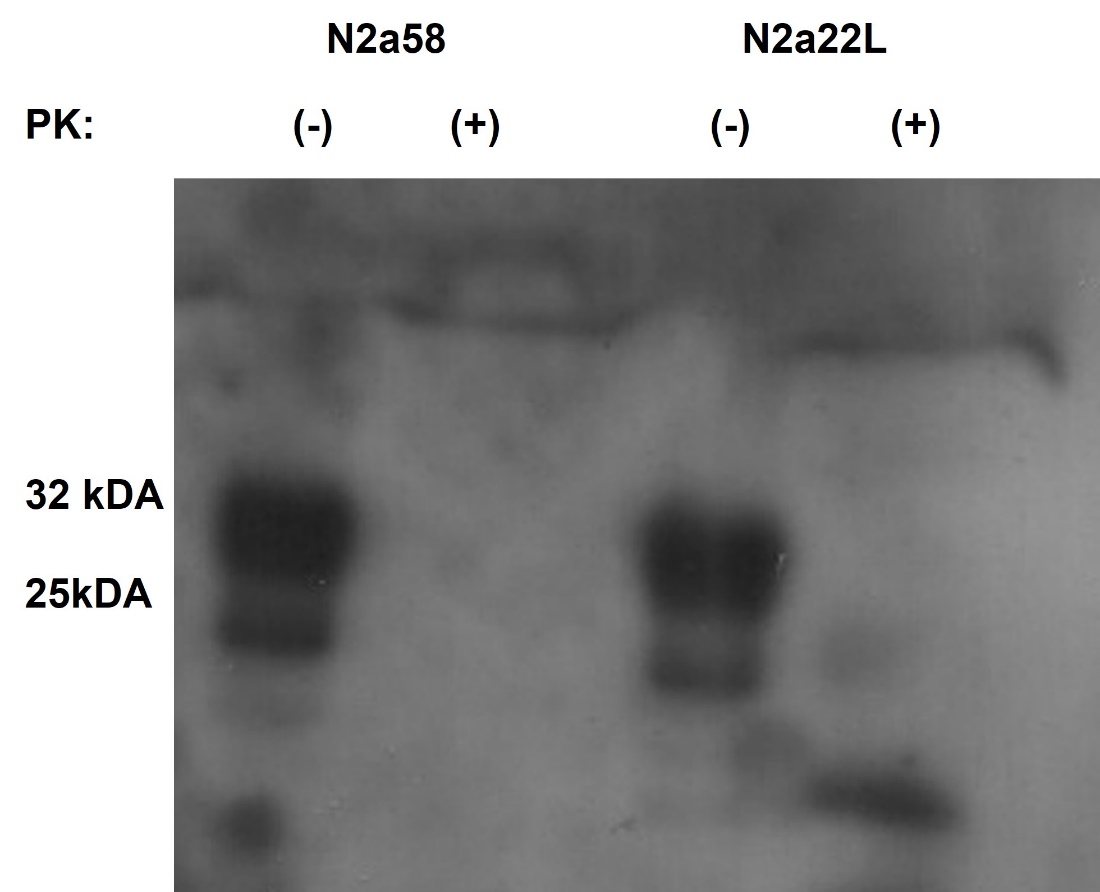


**Supplementary Figure 4:** Western blot of N2a58 and N2a22L cell lysates plus (+) or minus (-), treated with Proteinase K (PK+, 1.25 μg PK/ mg total protein) for 1 h at 37°C, to allow the PrP detection. In N2a58 cell fractions, PrP^C^ was detected in non- PK treated fractions, while it was completely digested following PK treatment. In N2a22L cell fractions, only the partially resistant PrP^Sc^ fraction was detected.
